# Fatigue and cognitive impairment in neuroborreliosis patients posttreatment—A neuropsychological retrospective cohort study

**DOI:** 10.1002/brb3.2719

**Published:** 2022-08-26

**Authors:** Anna Helena Sigurdardottir, Fredrikke Christie Knudtzen, Anita Nymark, Malcolm Bang

**Affiliations:** ^1^ Department of Psychology Faculty of Health Sciences University of Southern Denmark Odense Denmark; ^2^ Clinical Center of Emerging and Vector‐borne Infections Odense University Hospital Odense Denmark; ^3^ Department of Infectious Diseases Odense University Hospital Odense Denmark

**Keywords:** long‐term prognosis, neuroborreliosis, cognition, fatigue, treatment delay

## Abstract

**Background:**

The aim of this study was to determine the prevalence of fatigue and cognitive impairment in patients with neuroborreliosis (NB) posttreatment and to determine whether delayed treatment initiation led to higher levels of fatigue and cognitive impairment.

**Methods:**

The study population consisted of 88 patients with NB included between October 10, 2014, and August 21, 2020, at the Clinical Center for Emerging and Vector‐borne Infections at Odense University Hospital, Denmark. The Symbol Digit Modalities Test (SDMT) was used as a cognitive screening test, and the Modified Fatigue Impact Scale (MFIS) was used to assess the patients’ level of fatigue over the course of a year.

**Results:**

Overall, 14.3% of patients had an SDMT score indicative of cognitive impairment, and 38.8% of patients reported experiencing fatigue 12 months posttreatment. We found no statistically significant differences in fatigue or cognitive impairment when comparing the patients who had a treatment delay of ≤14 days and those with a treatment delay of >14 days (*p* > .05) 12 months posttreatment. A random effects regression model showed a significant positive correlation between longer treatment delay and higher MFIS scores, indicating higher levels of fatigue.

**Conclusions:**

The results of this study show that both the early and late treatment groups improved significantly over a 12‐month period in terms of both cognitive symptoms and fatigue. However, it also showed that a substantial subgroup of patients with NB still suffer from fatigue and cognitive impairment 12 months posttreatment.

## Graphical abstract

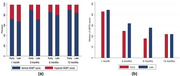

## 1 INTRODUCTION

Neuroborreliosis (NB), a tick‐borne infection caused by the spirochete *Borrelia burgdorferi*, is one of the most common central and peripheral nervous system infections in Europe (Obel et al., 2018). It occurs in 3%–15% of patients with borreliosis, and it typically manifests itself as meningoradiculitis, lymphocytic meningitis, and cranial nerve palsy (Dersch et al., 2016; Knudtzen et al., 2017). While most patients with NB recover fully from their symptoms, studies have shown that 10%–50% of patients report lasting symptoms and complaints such as cognitive problems, fatigue, and reduced health‐related quality of life (HRQoL) (Eikeland et al., 2012; Ljøstad & Mygland, 2010). Several studies have shown persisting complaints and symptoms in patients with NB even years after the end of treatment, with patients with NB displaying reduced attention, executive functioning, processing speed, verbal learning abilities, and memory (Eikeland et al., 2012, 2011; Treib et al., 2000). It has also been reported that many patients with NB experience increased levels of fatigue and reduced HRQoL long after their treatment has ended (Eikeland et al., 2011; Treib et al., 2000). Although it is not exactly known what causes these symptoms to persist after ending treatment, it is established that delayed treatment initiation is associated with residual symptoms and complaints persisting posttreatment in patients with NB (Knudtzen et al., 2017; Ljøstad & Mygland, 2010; Ritvo et al., 1997).

The aim of this retrospective cohort study was to determine the prevalence of fatigue and cognitive impairment in a well‐defined Danish cohort of patients with NB and to determine whether treatment delay (i.e., prolonged time from symptom debut to treatment initiation) increased the risk of persisting symptoms and complaints of fatigue and impaired cognitive functioning 1 year after treatment.

## 2 METHODS

### 2.1 Study design

This study was conducted as a retrospective cohort study of patients with NB who were followed at the Clinical Center of Emerging and Vector‐borne Infections (CCEVI) at Odense University Hospital (OUH), Denmark, in the time period from October 10, 2014 and August 21, 2020.

### 2.2 Study population and setting

All adult patients from CCEVI fulfilling the diagnostic criteria of definite or probable NB were eligible for inclusion. CCEVI is the referral center for all patients with suspected or confirmed NB in the Region of Southern Denmark with a population of approximately 1.2 million inhabitants (Statistics, Denmark. Population at the first day of the quarter by region, sex, age and marital status). Patients can be referred from general practitioners, private specialists, or other hospital departments.

### 2.3 Clinical data and background variables

Information regarding test scores from all patients was extracted from CCEVI clinical records. Information about age, sex, education, comorbidities using the Charlson Comorbidity Index (CCI) score (Quan et al., 2011), symptomology, time of symptom onset, and treatment initiation were gathered from the CCEVI database.

### 2.4 Outcome measures

The Symbol Digit Modalities Test (SDMT) was used as a cognitive screening test, and the Modified Fatigue Impact Scale (MFIS) was used to assess patients’ level of fatigue (Benedict et al., 2017; Ritvo et al., 1997). Both tests were administered at four different time points: 1, 3, 6, and 12 months posttreatment.

The SDMT consists of 120 symbols that are translated into numbers using a symbol translation key in the time frame of 90 s (Benedict et al., 2017). The raw score consists of the total number of correct answers within the given time frame. The raw score for the individual patient is also interpreted in light of the patient's age and educational level. This is done using normative data that allow converting this raw score into standard scores in the unit of standard deviations (SD) from the mean of the particular age and educational group to which the patient belongs. The SDMT categorizes the patients’ education into two groups: ≤12 years or >12 years of education (Benedict et al., 2017). We used the raw and standard scores in this study. For patients with poor or affected hand function, an oral version of the SDMT was administered. The SDMT has been shown to be particularly sensitive to measuring processing speed, which has been shown to account for impairments in executive functioning, working memory, learning, and memory in multiple sclerosis (MS) patients (Benedict et al., 2017).

The MFIS is a self‐report questionnaire that was originally developed to measure fatigue in patients with multiple sclerosis (Larson, 2013). It consists of 21 questions that measure three subscales of fatigue: physical fatigue, cognitive fatigue, and psychosocial fatigue. The respondents are asked to rate how often different scenarios have occurred to them within the last 4 weeks. It is rated on a Likert scale of 0–4, with 0 for never, 1 for rarely, 2 for sometimes, 3 for often, and 4 for almost always. The total score is calculated by adding all the ratings (Ritvo et al., 1997).

In addition to these measures, residual symptoms at the different time points posttreatment were registered from patient charts, including fatigue, concentration difficulties, memory difficulties, headache, radicular pain, and facial nerve palsy.

### 2.5 Definitions

Definite NB was defined as neurological symptoms consistent with NB, cerebrospinal fluid (CSF) pleocytosis, and a positive *B. burgdorferi* antibody index in the CSF (Mygland et al., 2010).

Probable NB was defined as relevant symptomology, CSF pleocytosis, an elevated CXCL13 > 50 ng/L, and clinical response to antibiotic therapy for NB (Knudtzen et al., 2020).

Treatment delay was defined as the number of days from the onset of neurological NB symptoms, as reported by the patients, until initiation of antibiotic treatment.

Cognition was measured by the SDMT, and we defined impaired cognition as patients scoring ≥1.5 SD below the mean. This cutoff was chosen based on studies showing that this cutoff correctly classified 92% of a group of adults with normal cognitive functioning and 86% of a group of adults with chronic brain lesions (Smith, 1973).

### 2.6 Grouping of patients

The included patients were analyzed as a whole in the descriptive analyses and in testing the difference in symptoms between the 1‐month follow‐up and the 12‐month follow‐up. In addition, patients were grouped based on the length of their treatment delay. A treatment delay of ≤14 days was categorized as the early treatment group, and a delay >14 days was categorized as the late treatment group.

### 2.7 Statistical methods

Statistical analyses were carried out using STATA version 16. Independent sample *t*‐tests were used for analyzing the difference between the groups for continuous data with a normal distribution, Wilcoxon Mann‒Whitney test and the Median test for non‐normally distributed data, and Pearson's *χ* ^2^‐test for categorical data. McNemar's test was used to analyze categorical data for the same group at two different times, and paired sample *t*‐tests and Wilcoxon signed‐rank tests were used to analyze continuous data for the same group at two different time points. A *p*‐value of .05 was considered significant.

Pearson's correlation coefficients were calculated, and random effects regression analyses were conducted to estimate the correlation between MFIS, SDMT, and treatment delay in days for the patient group as a whole.

The software G*Power 2 was used for subsequent statistical power analyses to estimate the statistical power of the included analyses with the available sample size.

### 2.8 Ethical considerations

The study was approved by the Danish Data Protection Agency (j.nr.16/31743) and the Danish National Committee on Health Research Ethics (Project‐ID S‐20160143).

## 3 RESULTS

### 3.1 Patient characteristics

The study population consisted of 88 patients, 80 with definite NB and eight with probable NB (Table 1). The median antibiotic treatment delay for the patients was 25 days (IQR: 14–53 days), 23 patients with a treatment delay of ≤14 days, and 65 patients with a treatment delay of >14 days.

**TABLE 1:** Characteristics of 88 patients with neuroblastoma treated at the Clinical Center of Emerging and Vector‐borne Infections, Odense, Denmark, between October 10, 2014 and August 21, 2020

Variables	Group as a whole *n* = 88	Early treatment group *n* = 23	Late treatment group *n* = 65	*p*‐Value
Age, years, median (IQR)	60 (48–69)	65 (47–70)	59 (48–68)	.676
Sex, female (%)	44 (50.0)	11 (47.8)	33 (50.8)	.808
LNB, definite (%)	80 (90.0)	18 (78.3)	62 (95.4)	.026
Charlson Comorbidity Index (%)				.751
0	64 (72.7)	16 (69.6)	48 (73.9)	
1–2	23 (26.2)	7 (30.4)	16 (24.6)	
≥3	1 (1.1)	0 (0.0)	1 (1.5)	
Education, year (%)				.007
≤12	29 (33.0)	11 (47.8)	18 (27.7)	
>12	59 (67.0)	12 (62.2)	47 (72.3)	

Abbreviations: IQR, interquartile range; LNB, Lyme neuroborreliosis.

Overall, 70 patients (79.6%) attended the 1‐month follow‐up, 75 (85.2%) attended the 3‐month follow‐up, 76 (86.4%) attended the 6‐month follow‐up, and 70 (79.6%) attended the 12‐month follow‐up. Forty‐three patients (48.9%) attended all four follow‐up visits.

### 3.2 Symptoms 12 months after the end of the treatment in patients with NB

As Table 2 shows, the SDMT was completed by 70 patients at the 12‐month follow‐up. The median SDMT score here was 43 (IQR: 36–50), and 14.3% obtained an SDMT score that was indicative of cognitive impairment adjusted for age and education level. This was a significant improvement from the first follow‐up at 1 month, where the median SDMT score was 37 (IQR: 29–45) (*p* < .001), and 31.4% obtained an SDMT score indicative of cognitive impairment (*p* = .012).

**TABLE 2:** Outcome measures in 88 patients at 1‐month follow‐up and 12‐month follow‐up after treatment for neuroborreliosis

Outcome measures	One month	12 months	*p*‐Value
	*n = 70*	*n = 70*	
SDMT raw score median (mean) (IQR)	37 (35.8)	43 (41.5)	<.001
	(29–45)	(36–50)	
Impaired cognition, No. (%)^a^	22 (31.4)	10 (14.3)	.012
	*n = 68*	*n = 59*	
MFIS score median (mean) (IQR)	44 (38.7)	22 (26.2)	<.001
	(26–53)	(11–39)	
	*n = 70*	*n = 67*	
Fatigue, No. (%)	55 (78.6)	26 (38.8)	<.001
Concentration difficulties, No. (%)	36 (51.4)	15 (22.4)	<.001
Memory difficulties, No. (%)	30 (42.9)	11 (16.7)	<.001
Radicular pain, No. (%)	58 (82.9)	4 (6.0)	<.001
Headache	19 (27.1)	4 (6.0)	<.001
Facial nerve palsy	34 (48.6)	3 (4.5)	<.001

*Note*: The exact McNemar's test and Wilcoxon signed‐rank test were used.

Abbreviations: IQR, interquartile range; MFIS, modified Fatigue Impact Scale; SDMT, symbol digit modalities test.

^a^ Impaired cognition defined as > 1.5 SD from the mean.

**FIGURE 1:**
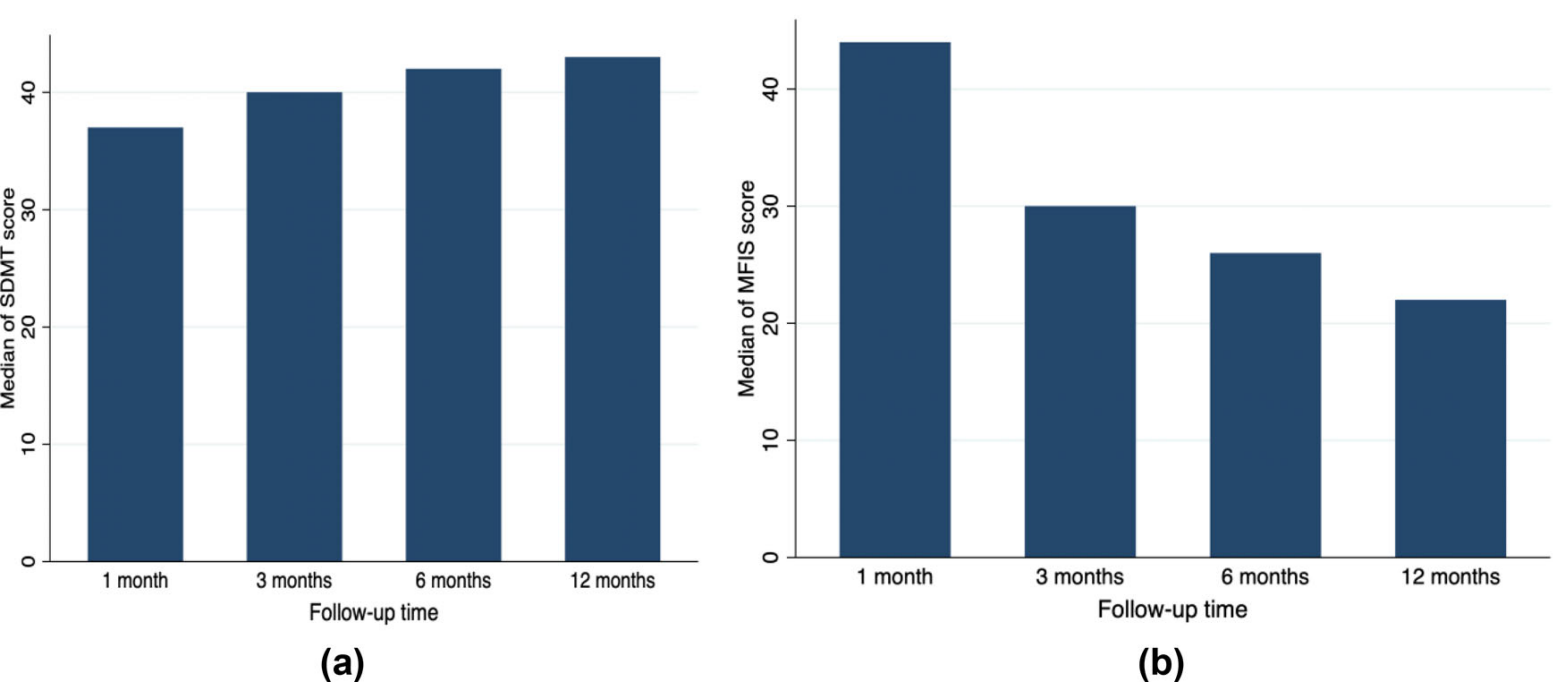
Median Symbol Digit Modalities Test (SDMT) score (a) and Modified Fatigue Impact Scale (MFIS) score (b) at four follow‐up times (1, 3, 6, and 12 months after the end of antibiotic treatment) in 88 patients with neuroborreliosis treated at the Clinical Center of Emerging and Vector‐borne Infections, Odense, Denmark, between October 10, 2014 and August 21, 2020. A lower SDMT score indicates a higher level of cognitive impairment, whereas a higher MFIS score indicates a higher level of fatigue

The SDMT score gradually increased over time, and at 3 and 6 months, the median scores were 40 (IQR 31–46) and 42 (IQR 34–49), respectively (Figure 1a) (Figure S1).

The MFIS was completed by 59 patients at the 12‐month follow‐up, where the median MFIS score was 22 (IQR: 11–39). This was significantly lower than the median MFIS score at the 1‐month follow‐up (median score 44 [IQR: 26‐53]) (*p* < .001).

The MFIS score gradually decreased over time, and at three and six months, the median scores were 30 (IQR 14–44) and 26 (IQR 12–39), respectively (Figure 1b) (Figure S2).

Twelve months after treatment, all of these symptoms were reported by significantly fewer patients (Table 2).

### 3.3 Difference in patient characteristics and treatment delay between the early and late treatment groups

We tested for differences in background variables between the early and late treatment groups and found no significant difference in terms of age, sex, CCI score, or educational level. The only significant difference found was that more patients fulfilled the definite NB criteria in the late treatment group (95.4%) than in the early treatment group (78.3%) (*p* = .026). This is shown in Table 1.

The early treatment group had a median treatment delay of 7 days (IQR: 4–12 days), and the late treatment group had a median treatment delay of 35 days (IQR: 24–62 days).

### 3.4 Difference between the early and late treatment groups in cognitive functioning and self‐reported symptoms at the 12‐month follow‐up

No significant differences in any of the self‐reported symptoms were found between the early and late treatment groups at the 12‐month follow‐up (Table 3). The largest difference in self‐reported symptoms between the groups was concentration difficulties, where 16.6% more patients in the late treatment group reported still displaying the symptom than in the early treatment group. Fatigue was reported in 10.1% more patients in the late group than in the early group (Table 3).

**TABLE 3:** Outcome measures at the 12‐month follow‐up in early versus late treatment groups of neuroborreliosis patients treated at the Clinical Center of Emerging and Vector‐borne Infections, Odense, Denmark, between October 10, 2014 and August 21, 2020

Outcome measures	Early treatment group	Late treatment group	*p*‐Value
	*n = 20*	*n = 50*	
SDMT score median (mean) (IQR)	42.5 (39.6)	43.5 (42.3)	.910
	(35.5–49)	(36–50)	
Impaired cognition, No. (%)*	2 (10)	8 (16)	.713
	*n = 16*	*n = 43*	
MFIS score median (mean) (IQR)	22 (24.6)	22 (26.8)	.831
	(12.5–39)	(10–39)	
	*n = 19*	*n = 48*	
Fatigue, No. (%)	6 (31.6)	20 (41.7)	.580
Concentration difficulties, No. (%)	2 (10.5)	13 (27.1)	.200
Memory difficulties, No. (%)	3 (15.8)	8 (17.0)	1.000
Radiculitis, No. (%)	0 (0)	4 (8.3)	.571
Headache, No (%)	0 (0)	4 (8.3)	.571
Palsy, No. (%)	1 (5.3)	2 (4.2)	1.000

*Note*: The Wilcoxon median test and Fisher's exact test, two‐tailed, were used to measure the significance level of differences.

Abbreviation: IQR, interquartile range.

* Defined as > 1.5 SD from the mean.

**FIGURE 2:**
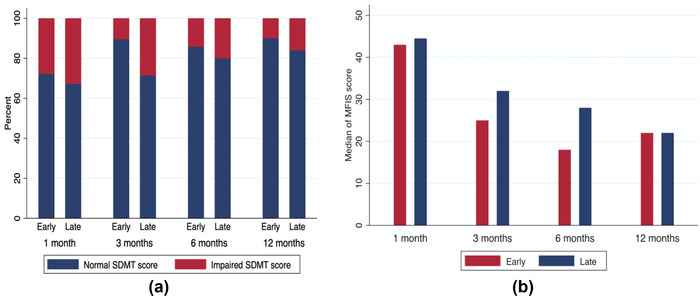
Percentage of impaired versus. normal Symbol Digit Modalities Test (SDMT) scores and median Modified Fatigue Impact Scale (MFIS) scores at four time points over 1 year in patients with early and late antibiotic treatment in 88 patients with neuroborreliosis treated at the Clinical Center of Emerging and Vector‐borne Infection, Odense University Hospital, Denmark, between October 10, 2014 and August 21, 2020. An SDMT score ≥1.5 SD from the mean is defined as indicative of cognitive impairment

At the 1‐month follow‐up, 27.8% of the early treatment group had impaired cognitive functioning as measured by the SDMT; at the 3‐month follow‐up, this decreased to 10.5%; at the 6‐month follow‐up, this increased to 14.3%; and at the 12‐month follow‐up, it decreased to 10.0%. (Figure 2a). In the late treatment group, the comparable numbers were 32.7% at the 1‐month follow‐up, 28.6% at three months, 20.0% at six months, and 16.0% at 12 months.

The largest difference in the number of patients with cognitive impairment between the two groups was seen at the 3‐month follow‐up, where 10.5% of the early treatment group displayed cognitive impairment, whereas 38.6% of the late treatment group displayed cognitive impairment. However, this difference was not found to be significant (*p* = .096). At the 12‐month follow‐up, the difference between the two groups’ median SDMT scores was 1, and there were 6% more patients with scores indicating impaired cognition in the late treatment group than in the early treatment group (*p* = .713).

### 3.5 Difference between the early and late treatment groups in the development of fatigue

The median MFIS score for the early treatment group was 43 (IQR 27–46) at the 1‐month follow‐up, at the 3‐month follow‐up it was 25 (IQR 16–38), at the 6‐month follow‐up it was 18 (IQR 8–36), and lastly at the 12‐month follow‐up it was 22 (IQR 13–39) (Figure 2b).

In the late treatment group, the median MFIS score at the 1‐month follow‐up was 45 (IQR 23–54), at three months it was 32 (IQR 14–48), at six months it was 28 (IQR 12–40), and finally at the 12‐month follow‐up 22 (IQR 10–39).

The largest difference in fatigue levels between the two groups was at the 6‐month follow‐up, where the median MFIS score was 10 points higher in the late treatment group than in the early treatment group. However, this difference was not found to be significant (*p* = .353), and at the 12‐month follow‐up, it had evened out, as illustrated in Table 3 and Figure 2b.

### 3.6 Statistical power analyses

As mentioned previously, the differences in symptoms between the early and late treatment groups at the 12‐month follow‐up were not found to be statistically significant (Table 3). Due to the small sample size and a suspicion of poor statistical power, post hoc statistical power analyses were conducted on the analyses testing differences between groups. These results showed that the included tests had limited statistical power. A priori analyses showed that the conducted *t*‐tests would have required a sample size of a minimum 191 patients to find a statistically significant effect size of 0.2, and the conducted *χ* ^2^‐test would have required a sample size of a minimum 197 patients to find a statistically significant effect size of 0.2.

### 3.7 Effects of treatment delay on SDMT and MFIS scores

To examine the effects of treatment delay (measured in days) on MFIS and SDMT scores in the patient group as a whole, a random effects regression analysis on panel data was conducted, using data from all four follow‐up times. A Houseman test showed that a random effects model was the preferred model for this analysis. This analysis showed that longer treatment delay was significantly positively correlated with higher MFIS scores when controlling for age, sex, and follow‐up time (*p *= .005) (Table 4). It showed a negative correlation between age and MFIS scores and a negative correlation between being male and MFIS scores. We also controlled for education in the model, but this was not found to be significant.

**TABLE 4:** The effects of treatment delay in days on patients’ Modified Fatigue Impact Scale (MFIS) score while controlling for age, sex, and follow‐up time

MFIS	Coefficient	SE	*p*‐Value
Treatment delay, days	0.022	0.008	.005
Sex, male	−7.832	3.571	.028
Age, years	−.291	0.121	.016
Follow‐up time, months	−1.069	0.175	<.001

Observations = 267.

*R* ^2 ^= 0.200.

A random effects regression analysis was also conducted with SDMT scores as the outcome variable, but this model did not find a significant correlation.

A Pearson's correlation coefficient analysis also showed treatment delay in days and MFIS scores at 12 months to be moderately positively correlated *r*(57) = 0.42, *p* < .001. This indicates that longer treatment delay is correlated with a higher level of fatigue. No significant correlation was found for treatment delay and SDMT scores (*p* = .378).

## 4 DISCUSSION

In this retrospective cohort study of patients with NB posttreatment, our most important findings were the large proportion of patients with fatigue and cognitive impairment after treatment for NB, the significant bettering over time on all parameters, and the fact that even though the progression was slower in patients with a longer delay from symptom debut to antibiotic treatment, the differences between the groups were not significant after 12 months. We also found that a longer treatment delay was associated with higher levels of fatigue as measured by the MFIS. An association was not found between treatment delay and cognitive functioning as measured by the SDMT.

Patients’ symptoms improved significantly over the year they were followed at the CCEVI, with the number of patients reporting fatigue, memory difficulties, and concentration difficulties decreasing gradually. These results were confirmed by a significant improvement in both cognitive functioning, measured with the SDMT, and fatigue, measured with the MFIS. These results indicate that the prognosis of the majority of patients with NB is generally good and that most patients with NB recover from their symptoms. It is important to note, however, that a substantial subgroup of patients do not recover to the same extent; 14.3% of patients still display cognitive impairment, 38.8% of patients still report being fatigued, and 22.4% still report having concentration difficulties 1 year posttreatment.

These results are similar to what has been found in other studies. Two Norwegian case‒control studies also found that the majority of patients with NB recover from their symptoms, but a subgroup of patients still have cognitive deficits and complaints of fatigue, concentration difficulties, and memory difficulties 30 months posttreatment (Eikeland et al., 2012, 2011). Similarly, a Swedish study of NB found that 25% of the patients still suffered from residual neurological symptoms five years posttreatment. They also indicated that early treatment and diagnosis could play an important role in the prognosis of patients (Berglund et al., 2002). Another study that conducted a range of neuropsychological tests on treated patients with NB found that although the mean cognitive functioning of the patients with NB was within the normal range, the patients with NB performed worse on tests assessing executive functioning compared to a control group (Schmidt et al., 2015).

We also noticed a difference in how the early treatment and late treatment groups fared in their SDMT and MFIS performances over the course of the four follow‐up times. The early treatment group improved faster than the late treatment group, indicating that a long treatment delay leads to a slower recovery. Both groups had similar levels of cognitive functioning and fatigue levels at 12 months. However, as the majority of patients with NB are previously healthy and of working age, the longer recovery time in this group may have substantial socioeconomic consequences (Henningsson et al., 2010).

It is also noteworthy that although only 14.3% of patients had cognitive impairment as measured by the SDMT at the 12‐month follow‐up, more patients reported experiencing cognitive symptoms such as concentration difficulties (22.4%) at this time. Additionally, although the mean MFIS score was relatively low at 25.2, 38.8% reported experiencing fatigue at the 12‐month follow‐up. These findings are similar to what has been found in a Norwegian study by Eikeland et al. (2011). They found that more patients reported memory problems, fatigue, and concentration difficulties than the control group, but these differences were not confirmed by their cognitive screening test.

Using random effects regression analysis, we found that longer treatment delay was significantly correlated with higher MFIS scores, indicating higher levels of fatigue. This was also shown using Pearson's correlation coefficient at the 12‐month follow‐up. Interestingly, the model also showed that being female was significantly correlated with higher levels of fatigue as measured by the MFIS and that being younger was also correlated with higher levels of fatigue.

When analyzing the groups based on a 14‐day cutoff, no significant difference was found, which may indicate that 14 days might not be the critical time point for treatment initiation.

A limitation of this study was its relatively small sample size, leading to reduced statistical power of the analyses that made it unlikely to find small effect sizes. We chose not to exclude patients who did not attend all four follow‐up visits. This may have influenced the results, as we do not know whether it was the patients who were doing well or the patients who were doing worse who did not attend all the follow‐up visits. The variation in symptoms and test performance at the four follow‐up times may be partially explained by this. Excluding all patients who did not attend all four follow‐up visits would have resulted in a substantially smaller sample size. This may also have resulted in attrition or selection bias due to potential differences between the patients who attended all four follow‐up visits and those who did not.

It is also important to note that we did not have a negative control group that was not followed at the CCEVI. The patients who were included in this study were all followed and received support and guidance at the clinic. Therefore, it is not possible for us to determine how patients who do not receive similar help might be affected.

We chose an arbitrary cutoff of 14 days post‐symptom debut to separate the early and late treatment groups. There is not much evidence in the literature that can point to a critical time frame that patients with NB must receive treatment for them not to be affected for long term. One previous study found that a treatment delay of more than 6 weeks in patients with NB was a potential risk factor for having residual symptoms and complaints 30 months after treatment (Eikeland et al., 2013; Tan et al., 2015).

The majority of the patients (90.9%) fulfilled the diagnostic criteria of definite NB, although there were fewer patients with definite NB in the early treatment group (78.3%). This is expected, as the *B. burgdorferi* antibody index’ sensitivity increases with time after symptom onset (Ljøstad et al., 2007; Mygland et al., 2010). In addition to classical NB symptoms and CSF pleocytosis, to fulfil the probable NB criteria, our patients had to present with an elevated CXCL‐13 score and respond positively to antibiotic therapy. This increases the probability of correct NB diagnoses and is a strength of the study, as many other studies have been criticized for not using strict diagnostic criteria in their study populations (Dersch et al., 2016).

Although studies have shown that the SDMT is a useful screening tool for cognitive impairment, its results should be interpreted with caution when used as the only measurement of cognitive functioning (Benedict et al., 2017; Smith, 1973). To obtain more valid and reliable results, the patients should be assessed with a battery of neuropsychological tests. Another important factor to consider when interpreting the SDMT results of this study is the potential practice effects that might occur, since the patients had to complete the test four times in the course of 1 year. One study of the SDMT found a significant practice effect in multiple sclerosis patients when they were tested on a monthly basis (Roar et al., 2016). However, this effect would probably be most likely to occur between the 1‐month and 3‐month follow‐up visits because of the shorter amount of time that passed between testing compared to the 12‐month follow‐up, where six months had passed since the patients were last tested.

It would also have been useful if data on population norms had been available for the MFIS to better determine whether the levels of fatigue experienced by the patients were higher than what is normal for the background population. We were also not able to find any studies validating the MFIS in Danish. This means that we do not know whether cultural bias could have affected the results. However, a study validating the scale in four different countries (Italy, Spain, Slovenia, and Belgium) found no cultural or linguistic differences in the psychometric properties of this scale (Kos et al., 2005).

It was not possible to know what the premorbid cognitive functioning of the patients was. We know, however, that the majority of the patient group (67%) were well educated with more than 12 years of education. We also know from another Danish study on patients with NB that before their diagnosis, patients with NB had higher employment rates and higher incomes and were less likely to receive disability pensions than a comparison cohort (Obel et al., 2018). Therefore, it is likely that the patient group had a level of cognitive functioning above average before being diagnosed with NB.

When considering the results of this study, it is important to note that the *B. burgdorferi* genospecies and the clinical manifestations in Europe and North American NB differ. Because of this, the results of this study may not be directly transferrable. The study population was representative of the general adult Danish NB population. The included patients were all referred to the CCEVI in the observation period on suspicion of or following diagnosis of NB, and all patients diagnosed with NB in the region were referred to this center, regardless of the severity of symptoms. Thus, there was a low risk of selection bias.

In conclusion, our findings showed that although the majority of patients with NB recovered fully within a year, 38.8% had one or more residual cognitive symptoms at this time. Our findings also indicated that patients in the early treatment group improved faster than those in the late treatment group and that longer treatment delay was associated with higher levels of fatigue. The study highlights the importance of increased awareness, early detection, and early treatment of NB.

## CONFLICTS OF INTEREST

All authors declare no conflicts of interest.

## AUTHOR CONTRIBUTIONS

*Design and conception*: Fredrikke Christie Knudtzen, Malcolm Bang, and Anna Helena Sigurdardottir. *Procurement of data*: Fredrikke Christie Knudtzen, Anita Nymark, and Anna Helena Sigurdardottir. *Interpretation of data*: Anna Helena Sigurdardottir, Fredrikke Christie Knudtzen, Malcolm Bang, and Anita Nymark. *Drafting of article*: Anna Helena Sigurdardottir, Fredrikke Christie Knudtzen, Malcolm Bang, and Anita Nymark. All authors read and approved the final version of the manuscript.

### PEER REVIEW

The peer review history for this article is available at https://publons.com/publon/10.1002/brb3.2719

## Supporting information

Supplementary Information

Supplementary Information

**Figure 3a and 3b**: Strip plot of SDMT scores (Figure 3a) and MFIS scores (Figure 3b) at four follow‐up times (1, 3, 6 and 12 months after ended antibiotic treatment) in 88 Patients with Neuroborreliosis treated at the Clinical Center of Emerging and Vector‐borne Infections, Odense, Denmark, between the 10^th^ of October 2014 and the 21^st^ of August 2020. A lower SDMT score indicates a higher level of cognitive impairment, whereas a higher MFIS score indicates a higher level of fatigue.

## Data Availability

The data that support the findings of this study are available on request from the corresponding author. The data are not publicly available due to privacy or ethical restrictions.

## References

[brb32719-bib-0001] Benedict, R. H. , DeLuca, J. , Phillips, G. , LaRocca, N. , Hudson, L. D. , Rudick, R. , & Multiple Sclerosis Outcome Assessments Consortium . (2017). Validity of the symbol digit modalities test as a cognition performance outcome measure for multiple sclerosis. Multiple Sclerosis Journal, 23(5), 721–733. 28206827 10.1177/1352458517690821 PMC5405816

[brb32719-bib-0002] Berglund, J. , Stjernberg, L. , Ornstein, K. , Tykesson‐Joelsson, K. , & Walter, H. (2002). 5‐y Follow‐up study of patients with neuroborreliosis. Scandinavian Journal of Infectious Diseases, 34(6), 421–425. 12160168 10.1080/00365540110080421

[brb32719-bib-0003] Dersch, R. , Sommer, H. , Rauer, S. , & Meerpohl, J. (2016). Prevalence and spectrum of residual symptoms in Lyme neuroborreliosis after pharmacological treatment: A systematic review. Journal of Neurology, 263(1), 17–24. 26459093 10.1007/s00415-015-7923-0

[brb32719-bib-0004] Eikeland, R. , Ljøstad, U. , Mygland, Å. , Herlofson, K. , & Løhaugen, G. (2012). European neuroborreliosis: Neuropsychological findings 30 months post‐treatment. European Journal of Neurology, 19(3), 480–487. 21999112 10.1111/j.1468-1331.2011.03563.x

[brb32719-bib-0005] Eikeland, R. , Mygland, Å. , Herlofson, K. , & Ljøstad, U. (2011). European neuroborreliosis: Quality of life 30 months after treatment. Acta Neurologica Scandinavica, 124(5), 349–354. 21303350 10.1111/j.1600-0404.2010.01482.x

[brb32719-bib-0006] Eikeland, R. , Mygland, Å. , Herlofson, K. , & Ljøstad, U. (2013). Risk factors for a non‐favorable outcome after treated E uropean neuroborreliosis. Acta Neurologica Scandinavica, 127(3), 154–160. 22690926 10.1111/j.1600-0404.2012.01690.x

[brb32719-bib-0007] Henningsson, A. , Malmvall, B.‐E. , Ernerudh, J. , Matussek, A. , & Forsberg, P. (2010). Neuroborreliosis—An epidemiological, clinical and healthcare cost study from an endemic area in the south‐east of Sweden. Clinical Microbiology and Infection, 16(8), 1245–1251. 19793326 10.1111/j.1469-0691.2009.03059.x

[brb32719-bib-0008] Knudtzen, F. C. , Andersen, N. S. , Jensen, T. G. , & Skarphédinsson, S. (2017). Characteristics and clinical outcome of Lyme neuroborreliosis in a high endemic area, 1995–2014: A retrospective cohort study in Denmark. Clinical Infectious Diseases, 65(9), 1489–1495. 29048514 10.1093/cid/cix568

[brb32719-bib-0009] Knudtzen, F. C. , Nilsson, A. C. , Hovius, J. W. , & Skarphedinsson, S. (2020). The predictive value of CXCL13 in suspected Lyme neuroborreliosis: A retrospective cross‐sectional study. European Journal of Clinical Microbiology & Infectious Diseases, 39, 1461–1470. 32172371 10.1007/s10096-020-03861-4

[brb32719-bib-0010] Kos, D. , Kerckhofs, E. , Carrea, I. , Verza, R. , Ramos, M. , & Jansa, J. (2005). Evaluation of the modified fatigue impact scale in four different European countries. Multiple Sclerosis Journal, 11(1), 76–80. 15732270 10.1191/1352458505ms1117oa

[brb32719-bib-0011] Larson, R. D. (2013). Psychometric properties of the modified fatigue impact scale. International Journal of MS Care, 15(1), 15–20. 24453758 10.7224/1537-2073.2012-019 PMC3883028

[brb32719-bib-0012] Ljøstad, U. , & Mygland, Å. (2010). Remaining complaints 1 year after treatment for acute Lyme neuroborreliosis; frequency, pattern and risk factors. European Journal of Neurology, 17(1), 118–123. 19645771 10.1111/j.1468-1331.2009.02756.x

[brb32719-bib-0013] Ljøstad, U. , Skarpaas, T. , & Mygland, Å. (2007). Clinical usefulness of intrathecal antibody testing in acute Lyme neuroborreliosis. European Journal of Neurology, 14(8), 873–876. 17662007 10.1111/j.1468-1331.2007.01799.x

[brb32719-bib-0014] Mygland, Å. , Ljøstad, U. , Fingerle, V. , Rupprecht, T. , Schmutzhard, E. , & Steiner, I. (2010). EFNS guidelines on the diagnosis and management of European Lyme neuroborreliosis. European Journal of Neurology, 17(1), 8–e4. 19930447 10.1111/j.1468-1331.2009.02862.x

[brb32719-bib-0015] Obel, N. , Dessau, R. B. , Krogfelt, K. A. , Bodilsen, J. , Andersen, N. S. , Møller, J. K. , Roed, C. , Omland, L. H. , Christiansen, C. B. , Ellermann‐Eriksen, S. , Bangsborg, J. M. , Hansen, K. , Benfield, T. L. , Rothman, K. J. , Sørensen, H. T. , Andersen, C. Ø. , & Lebech, A.‐M. (2018). Long term survival, health, social functioning, and education in patients with European Lyme neuroborreliosis: Nationwide population based cohort study. BMJ, 361, k1998. 29848547 10.1136/bmj.k1998 PMC5974636

[brb32719-bib-0016] Quan, H. , Li, B. , Couris, C. M. , Fushimi, K. , Graham, P. , Hider, P. , Januel, J.‐ M. , & Sundararajan, V. (2011). Updating and validating the Charlson comorbidity index and score for risk adjustment in hospital discharge abstracts using data from 6 countries. American Journal of Epidemiology, 173(6), 676–682. 21330339 10.1093/aje/kwq433

[brb32719-bib-0017] Ritvo, P. G. , Fischer, J. S. , Miller, D. M. , Andrews, H. , Paty, D. , & LaRocca, N. (1997). Multiple sclerosis quality of life inventory: A user's manual. New York: National Multiple Sclerosis Society, 1997, 1–65.

[brb32719-bib-0018] Roar, M. , Illes, Z. , & Sejbaek, T. (2016). Practice effects in symbol digit modalities test in multiple sclerosis patients treated with natalizumab. Multiple Sclerosis and Related Disorders, 10, 116–122. 27919477 10.1016/j.msard.2016.09.009

[brb32719-bib-0019] Schmidt, H. , Djukic, M. , Jung, K. , Holzgraefe, M. , Dechent, P. , von Steinbüchel, N. , Blocher, J. , Eiffert, H. , & Schmidt‐Samoa, C. (2015). Neurocognitive functions and brain atrophy after proven neuroborreliosis: A case‐control study. BMC Neurology, 15(1), 139. 26286440 10.1186/s12883-015-0386-1 PMC4545711

[brb32719-bib-0020] Smith, A. (1973). Symbol digit modalities test. Western Psychological Services.

[brb32719-bib-0021] Statistics Denmark . Population at the first day of the quarter by region, sex, age and marital status. Secondary Statistics Denmark. Population at the first day of the quarter by region, sex, age and marital status. https://www.statistikbanken.dk/statbank5a/selectvarval/saveselections.asp

[brb32719-bib-0022] Tan, Y. C. , Gill, A. K. , & Kim, K. S. (2015). Treatment strategies for central nervous system infections: An update. Expert Opinion on Pharmacotherapy, 16(2), 187–203. 25328149 10.1517/14656566.2015.973851

[brb32719-bib-0023] Treib, J. , Grauer, M. T. , Haass, A. , Langenbach, J. , Holzer, G. , & Woessner, R. (2000). Chronic fatigue syndrome in patients with Lyme borreliosis. European Neurology, 43(2), 107–109. 10686469 10.1159/000008144

