# Supplementary figures and images for: Fatigue and cognitive impairment in neuroborreliosis patients posttreatment—A neuropsychological retrospective cohort study
Source: Brain Behav. 2022 Aug 26;12(9):e2719. doi: 10.1002/brb3.2719 (PMC9480899; doi:10.1002/brb3.2719)

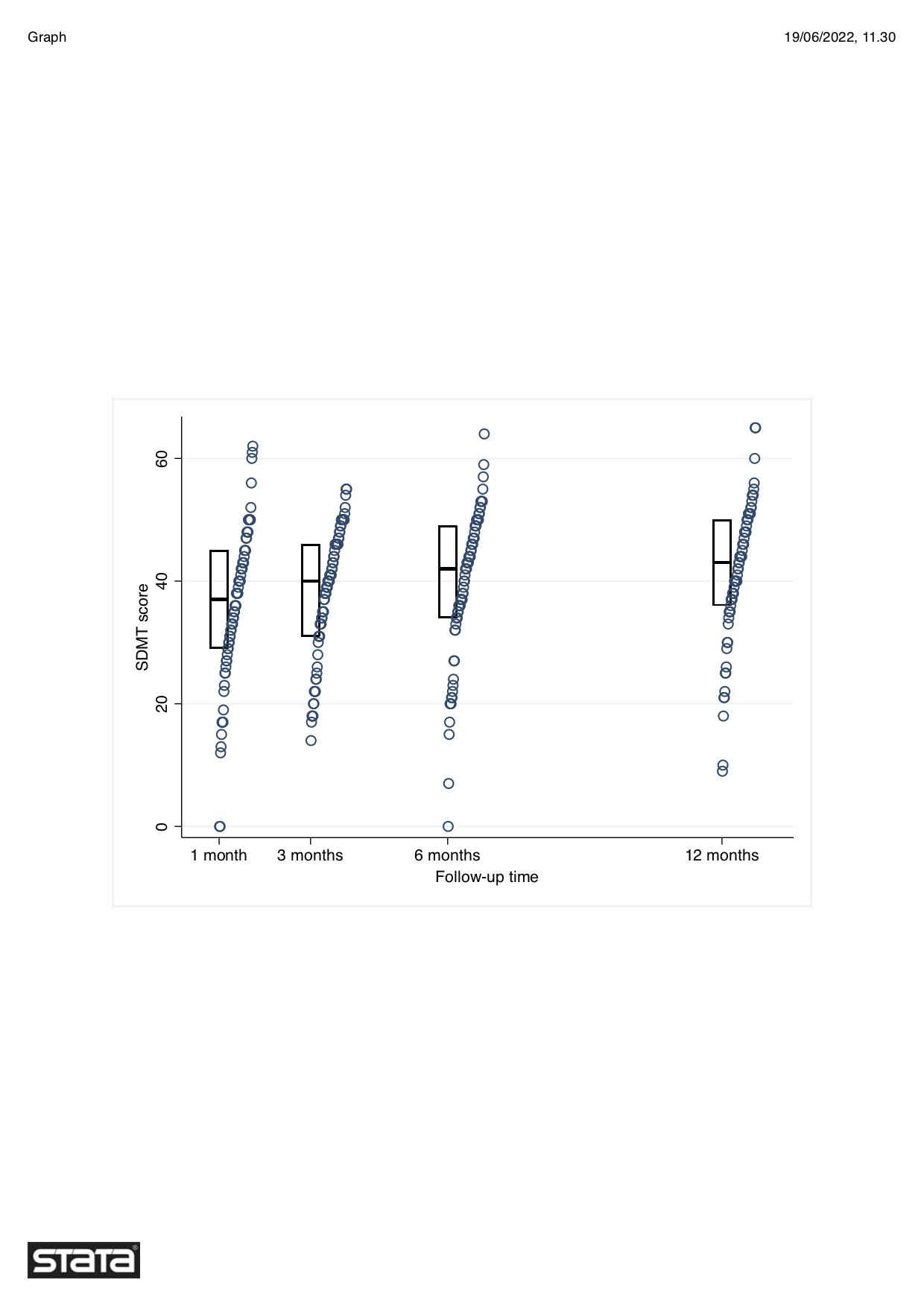

Supplement: Supplementary file 1 — Supplementary Information [file BRB3-12-e2719-s003.tiff]

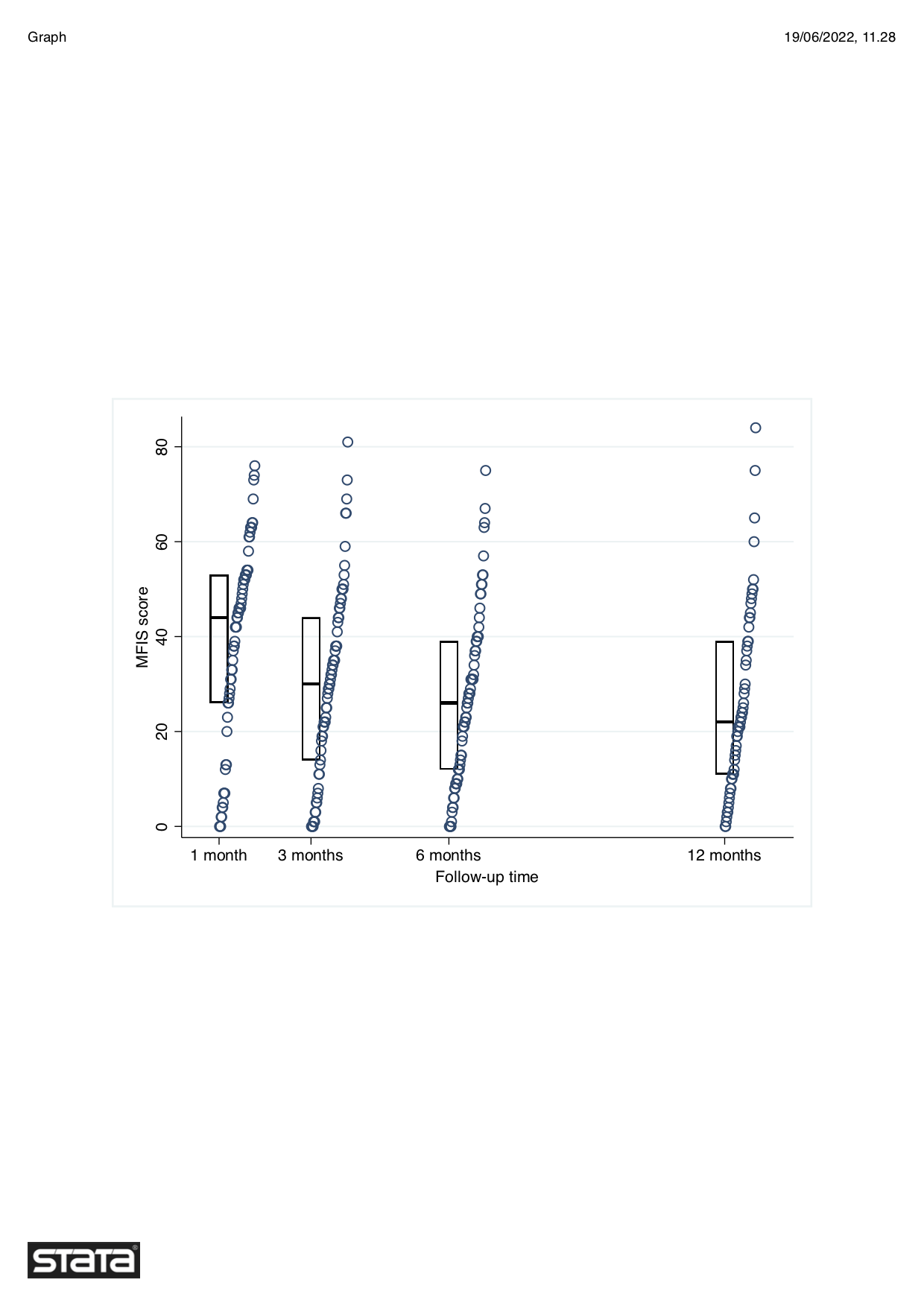

Supplement: Supplementary file 2 — Supplementary Information [file BRB3-12-e2719-s001.tiff]
