# Supplementary material for: Fatigue and cognitive impairment in neuroborreliosis patients posttreatment—A neuropsychological retrospective cohort study
Source: Brain Behav. 2022 Aug 26;12(9):e2719. doi: 10.1002/brb3.2719 (PMC9480899; doi:10.1002/brb3.2719)
Supplement: Supplementary file 3 — Figure 3a and 3b: Strip plot of SDMT scores (Figure 3a) and MFIS scores (Figure 3b) at four follow‐up times (1, 3, 6 and 12 months after ended antibiotic treatment) in 88 Patients with Neuroborreliosis treated at the Clinical Center of Emerging and Vector‐borne Infections, Odense, Denmark, between the 10th of October 2014 and the 21st of August 2020. A lower SDMT score indicates a higher level of cognitive impairment, whereas a higher MFIS score indicates a higher level of fatigue. [file BRB3-12-e2719-s002.docx]

**Supplementary material**

**Figure 3a and 3b:** *Strip plot of SDMT scores (Figure 3a) and MFIS scores (Figure 3b) at four follow-up times (1,3,6 and 12 months after ended antibiotic treatment) in 88 Patients with Neuroborreliosis treated at the Clinical Center of Emerging and Vector-borne Infections, Odense, Denmark, between the 10^th^ of October 2014 and the 21^st^ of August 2020.* *A lower SDMT score indicates a higher level of cognitive impairment, whereas a higher MFIS score indicates a higher level of fatigue.*
